# Effect of different dietary arachidonic, eicosapentaenoic, and docosahexaenoic acid content on selected immune parameters in gilthead sea bream (*Sparus aurata*)

**DOI:** 10.1016/j.fsirep.2021.100014

**Published:** 2021-06-24

**Authors:** R. Magalhães, F.A. Guardiola, I. Guerreiro, F. Fontinha, S. Moutinho, R.E. Olsen, H. Peres, A. Oliva-Teles

**Affiliations:** a Interdisciplinary Centre of Marine and Environmental Research (CIIMAR), University of Porto, Terminal de Cruzeiros do Porto de Leixões, Av. General Norton de Matos s/n, 4450-208 Matosinhos, Portugal; b Department of Biology, Faculty of Sciences of the University of Porto, Rua do Campo Alegre s/n, Ed. FC4, 4169-007 Porto, Portugal; c Department of Cell Biology and Histology. Faculty of Biology, Campus Regional de Excelencia Internacional “Campus Mare Nostrum”, University of Murcia, 30100, Murcia, Spain; d Department of Biology, Norwegian University of Science and Technology, Trondheim N-7491, Norway

**Keywords:** Arachidonic acid, Docosahexaenoic acid, Eicosapentaenoic acid, Haematology, Innate immunity

## Abstract

The increasing replacement of fish oil (FO) by vegetable oils (VO) in aquafeeds for marine carnivorous species may lead to modifications of the dietary arachidonic (ARA), eicosapentaenoic (EPA) and docosahexaenoic (DHA) levels and ratios that may compromise the fish immune status. Therefore, this study aimed to assess how different dietary ratios of ARA, EPA, and DHA modulate the immune response of gilthead sea bream juveniles. For that purpose, four isoproteic and isolipidic plant-based diets were formulated to include different dietary Long-Chain-Polyunsaturated Fatty Acids (LC-PUFA) ratios (%DM): Diet A (2.0% ARA: 0.2% EPA: 0.1% DHA); Diet B (1.0% ARA: 0.4% EPA: 0.4% DHA); Diet C (0% ARA: 0.6% EPA: 0.6% DHA); Diet D (0% ARA: 0.3% EPA: 1.5% DHA). Fish were fed the experimental diets for 56 days and then the haematological profile, humoral innate immune parameters, and expression of immune-related genes in the gut were determined. Our results showed that fish fed diet B exhibited an increase in the monocyte numbers, alternative complement activity, and bactericidal activity against *Photobacterium damselae* compared to the values found in fish fed other diets. Contrarily, peroxidase, antiproteases, and proteases activity and nitric oxide levels were not affected by the dietary treatments. Immune-related gene expression in the distal intestine did not show differences between dietary treatments. Overall, a diet with a balanced n-6 (ARA) and n-3 (EPA+DHA) LC-PUFA ratio seemed to better modulate the fish innate immune response, thus better preparing the fish innate immune system against potential immunological insults*.*

## Highlights

• A balanced dietary ARA/EPA/DHA level increased circulating monocytes numbers.
• A balanced dietary ARA/EPA/DHA level enhanced the haemolytic complement activity.
• Bactericidal activity against *P. damselae* was increased in fish fed Diet B (1.0% DM ARA: 0.4% DM EPA: 0.4% DM DHA).
• Optimum dietary ARA/EPA/DHA ratio better modulate the fish innate immune system.
• Immune-related gene expression in the distal intestine did not show differences between different dietary ARA/EPA/DHA levels.

## Graphical abstract

**Figure:**
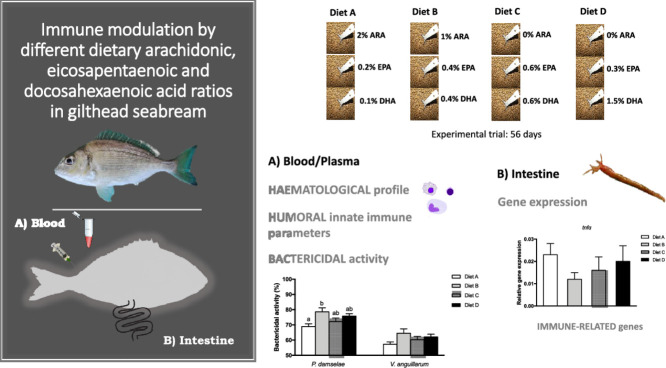

## 1 Introduction

Vegetable oils (VO) have been increasingly used as a more sustainable source of energy for aquafeeds [1] and are regarded as the major alternative for fish oil (FO) replacement in the diets due to their steady availability and lower price. However, in contrast to FOs, which are rich in LC-PUFA, namely eicosapentaenoic (EPA, 20:5n-3) and docosahexaenoic (DHA, 22:6n-3) acids, VOs are devoid of Long-Chain-Polyunsaturated Fatty Acids (LC-PUFA) [2, 3]. LC-PUFA are essential for marine fish since they have limited expression of Δ 6 and 5 desaturases, which are necessary to synthesize LC-PUFA from their C18-PUFA precursors [4]. This difficult meeting the essential fatty acids (EFA) requirements of marine fish species using VO as the major dietary lipid source [5]. Nevertheless, different VO blends have been used in the diets to replace FO at different levels in several marine fish species without loss of growth performance, proving that the EFA requirements are met [6]. For gilthead sea bream (*Sparus aurata*), 66 to 69 % of FO substitution by VO blend, soybean oil, or rapeseed oil has been achieved without significant effects on growth performance [7, 8].

The total n-3 LC-PUFA requirement for gilthead sea bream is still not adequately estimated and requirements of 0.9 or 1.9 % were reported for 42 g and 1 g fish, respectively [9, 10]. Further, the dietary DHA/EPA ratio of the diets was 1 and 0.5, which further complicates defining requirements as the optimum dietary n-3/n-6 ratio is still not defined. ARA requirement for gilthead sea bream juveniles was not yet established, but no variations in growth performance and feed utilization seem to occur with dietary ARA levels ranging from 0 to 1.7 % [11].

Besides growth, variations in the dietary n-3/n-6 fatty acids (FA) ratio due to the use of VO may have a significant impact on fish health, since many physiological functions are directly dependent on the dietary balance of these FA [4, 12]. For instance, the FA composition of cell membranes is affected by dietary lipids and can affect the immune cell FA composition [13], [14], [15]. Therefore, it can modulate the immune response in fish [14, 16] by modulating the physical properties of cell membranes and membrane-associated enzymes and receptor sites [17], immune cells functionality (i.e. phagocytosis and respiratory burst activity) [13, [18], [19], [20]], humoral defenses (i.e. lysozyme and alternative complement activity) and eicosanoid production [18, 19, 21, 22].

However, the mechanisms by which EFA modulates fish immune response are still not fully understood. Arachidonic acid content in the cell membrane is a substrate for cyclooxygenase-2 (Cox_2_) and 5-lipoxygenase (5-LOX) enzymes, that synthesize eicosanoids such as prostaglandin E2 (PGE_2_) and 4-series leukotrienes that promotes leucocytes chemotaxis, reactive oxygen species formation, and other pro-inflammatory effects [23]. Contrarily, n-3 LC-PUFA are known to compete with ARA as a substrate for the mentioned enzymes to produce less inflammatory eicosanoids such as PGE_3_ and 5-series leukotrienes and reducing the pro-inflammatory ARA derivates [24, 25]. Furthermore, ARA, EPA, and DHA may also regulate immune-related transcription factors such as nuclear factor kappa B (NF*_k_*B) which is a potent inducer of proinflammatory cytokine production [26]. Therefore, alterations in the cell membrane EFA profile of different immune cell types in circulation or specific tissues such as the distal intestine, which is characterized by a diffused presence of leucocytes in lamina propria and epithelium[27], may result in an inadequate immune response or humoral protection.

For gilthead sea bream, it has already been shown that the dietary FA profile has a significant impact on the humoral immune response [17, 21]. For instance, 1.5 % of dietary n-3 LC-PUFA was found to be necessary for the maintenance of alternative complement activity, especially at high stocking density [21], while a dietary decrease from 2 to 0.5 % of n-3 LC-PUFA reduced the alternative complement activity and the number of lymphocytes [17]. Further, the high dietary n-6 FA levels due to the replacement of FO by soybean oil induced a reduction of the serum bactericidal capacity against *Flavobacterium psychrophilum* [28]. Given the increased use of VO in aquafeeds, the purpose of this study was to assess the modulation of dietary n-6 /n-3 LC-PUFA ratio on the humoral innate immune and distal intestine inflammatory responses of gilthead sea bream juveniles.

## 2 Material and methods

### 2.1 Diets composition

Four practical diets were formulated to be isoproteic (47 % crude protein) and isolipidic (18 % crude lipids) containing fish meal (FM) and plant feedstuffs (PF) (26:74 protein from FM: PF) as main protein sources and a VO blend (20:50:30 rapeseed, linseed, and palm oils) as lipid source. The diets were supplemented EFA to obtain n6/n3 LC- PUFA ratios (%DM) of 2.0 ARA: 0.2 EPA: 0.1 DHA (Diet A); 1.0 ARA: 0.4 EPA: 0.4 DHA (Diet B); 0 ARA: 0.6 EPA: 0.6 DHA (Diet C); 0 ARA: 0.3 EPA: 1.5 DHA (Diet D). The commercial products used as EFA sources were the following: for ARA, Vevodar® (DSM Food Specialties, the Netherlands); for EPA, SuperbaKrill^TM^ Oil, Solchem®; and for DHA tuna oil (70 % DHA; BrudyTechnology®).

All diet ingredients were thoroughly mixed and dry pelleted in a laboratory pellet mill (California Pellet Mill, CPM Crawfordsville, IN, USA) through 3.0 mm die. Pellets were dried in an oven at 40°C for 48 h and kept in a freezer in airtight bags until use. Proximate composition of the ingredients and experimental diets and fatty acid profiles are presented in Magalhães *et al.* [29] and dietary EFA profiles are summarized in Table 1.

**Table 1:** Essential fatty acid composition and n-6/n-3 ratio of the experimental diets (% of total fatty acids).

Diets	A	B	C	D
(ARA/EPA/DHA ratio)	2.0/0.2/0.1	1.0/0.4/0.4	0/0.6/0.6	0/0.3/1.5
20:4n-6 (ARA)	10.6	5.43	0.18	0.11
20:5n-3 (EPA)	0.96	2.02	3.24	1.70
22:6n-3 (DHA)	0.70	2.06	3.52	7.71
n-6/n-3	1.50	0.98	0.63	0.52

ARA: Arachidonic; EPA: eicosapentaenoic; DHA: docosahexaenoic

### 2.2 Experimental design

The experiment was conducted in CIIMAR, Matosinhos, Portugal, with gilthead sea bream juveniles (15 g) obtained from a commercial fish farm (Maresa S.A., Ayamonte, Huelva, Spain). The trial was performed in a recirculating aquatic system (RAS) thermo-regulated to 23.0 ± 1.0°C and equipped with 12 cylindrical fiberglass tanks of 100 L water capacity. Tanks were maintained with a continuous flow of filtered seawater (2.5–3.5 L min^−1^), salinity (35 ± 1 g L^−1^) and dissolved oxygen near saturation (7 mg L^−1^). A photoperiod of 12 hours light and 12 hours of darkness was established. Fish were transferred to the experimental system after a quarantine period of one month and adapted to the experimental conditions for 15 days and fed with a commercial diet (NEO, Aquasoja). Then, twenty fish were distributed to each tank, and the experimental diets aleatory assigned to triplicate groups. Fish were fed by hand, twice daily, 6 days a week, until apparent visual satiation for 56 days. Care was taken to avoid waste of feed. The experiment was approved by CIIMAR ethical committee for Managing Animal Welfare (ORBEA), in compliance with the European Union directive 2010/63/EU and the Portuguese Law (DL 113/2013).

### 2.3 Sampling

Three fish from each tank were randomly collected 4 hours after the morning meal for blood. Blood samples were withdrawn from the caudal vein with heparinized syringes and utilized for total erythrocytes and leucocytes counts. Then blood smears were prepared. The remaining blood was immediately centrifuged (10,000 *× g* for 10 min, room temperature) and plasma collected and stored at -80°C until further analyses. Following blood collection, the fish were euthanized by decapitation and the intestine was dissected on chilled trays. The digestive tract was freed from adjacent adipose and connective tissues and a portion of the distal intestine (DI; distinguished from the mid intestine by an enlarged diameter and darker mucosa) from two fish per tank were sampled and then stored for gene expression analysis.

### 2.4 Chemical analysis

Chemical analysis of the diets was performed according to the Association of Official Analytical Chemists methods AOAC [30]. Briefly, the dry matter was assessed by drying the samples in an oven at 105˚ C until constant weight and ash content by incineration in a muffle furnace at 450˚C for 16 h. The Kjeldahl method was used for the determination of protein content (N x 6.25) using a Kjeltec digestion and distillation units (Tecator Systems; models 1015 and 1026, respectively). Crude lipids were determined using petroleum ether as a solvent and a Soxtec HT System (Höganäs); dietary starch was determined according to Beutler [31]. Gross energy was quantified by direct combustion in an adiabatic bomb calorimeter (PARR model 6200; Parr Instruments). FA quantification was done according to Magalhães *et al.* [29].

### 2.5 Blood parameters

Blood parameters were accessed according to Peres *et al.* [32]. Briefly, total white (WBC) and red (RBC) blood cell count was made from blood dilutions using a hemocytometer. Fresh heparinized blood was used for haematocrit (HT) and its value was determined by the micro centrifugation (10,000 x *g* for 10 min, at room temperature) of haematocrit tubes. Drabkin´s solution was used for haemoglobin determination (HB; SPINREACT kit, ref. 1,001,230, Spain). The mean corpuscular haemoglobin (MCH), mean corpuscular volume (MCV), and mean corpuscular haemoglobin (MCHC) were calculated as follows:

• MCH (pg cell^−1^) = (HB/RBC) x 10
• MCV (µm^3^) = (HT/RBC) x 10
• MCHC (g 100 mL^−1^) = (HB/HT) x 100

Immediately after blood collection, blood smears were made from fresh blood, air-dried, fixed with formol-ethanol (3.7% formaldehyde in absolute ethanol), and stained with Wright's solution (Haemacolor; Merck). Neutrophils were detected by their peroxidase activity as described by Afonso *et al.* [33]. The slides were carefully analyzed under oil immersion (1,000x), and at least 200 leucocytes were counted and classified as thrombocytes, lymphocytes, monocytes, and neutrophils. The absolute value (x 10^4^ µL^−1^) of each cell type was calculated.

### 2.6 Humoral innate immune parameters

#### 2.6.1 Protease activity

Protease activity was quantified using the azocasein hydrolysis according to Guardiola *et al.* [34] with some modifications. After adding 110 µL of 0.1 M phosphate buffer (pH 7.0) and 125 µL of 2 % azocasein (Sigma) to 10 µL of plasma, samples were incubated for 24 h at 22 ˚C and, following the addition of 250 µL of 10 % trichloroacetic acid (TCA), were incubated for 30 min at 22 ˚C. The mixture was then centrifuged at 10,000 *x g* for 5 min. Supernatants (100 µL) were transferred to 96-well plates containing 100 µL well^−1^ of 1 N NaOH and the optical density (OD) was read at 450 nm using a microplate reader (Synergy HT). Plasma was replaced by trypsin (5 mg mL^−1^, Sigma) as a reference sample (100% of protease activity), or by a buffer as blank (0% activity).

#### 2.6.2 Antiprotease activity

Total antiprotease activity was determined by the ability of plasma to inhibit trypsin activity according to Machado *et al.* [35]. Briefly, 10 µL of each plasma sample was incubated with the same volume of standard trypsin solution (Sigma, 5 mg mL^−1^) for 10 min at 22 ˚C. After adding 100 µL of 0.1 M phosphate buffer (pH 7.0) and 125 µL of 2 % azocasein (Sigma), samples were incubated for 1 h at 22 ˚C and, following the addition of 250 µL of 10 % TCA, were incubated for 30 min at 22 ˚C. The mixture was then centrifuged at 10,000 *x g* for 5 min. Supernatants (100 µL) were transferred to 96-well plates containing 100 µL well^−1^ of 1 N NaOH, and the optical density was read at 450 nm using a microplate reader (Synergy HT). Phosphate buffer instead of plasma and trypsin served as blank whereas the reference sample was phosphate buffer instead of plasma. The percentage of inhibition of trypsin activity was calculated by comparison with the reference sample.

#### 2.6.3 Peroxidase activity

The peroxidase activity was measured according to Quade and Roth [36]. Briefly, 5 µL of plasma was diluted with 40 µL of Hank's buffer (HBSS) without Ca^+2^ or Mg^+2^ in flat-bottomed 96-well plates. 100 µL of 10 mM 3,3’,5,5’- tetramethylbenzidine hydrochloride (TMB, Sigma-Aldrich), and 0.015% H_2_O_2_ were used as substrates. The colorimetric reaction was concluded after 2 min by adding 50 µL of 2 M sulphuric acid (H_2_SO_4_) and the OD was read at 450 nm in a microplate reader (Synergy HT). HBSS instead of plasma was used as blank. The peroxidase activity was calculated by defining 1 OD absorbance change as corresponding to 1 unit of peroxidase activity (units mL^−1^ of plasma).

#### 2.6.4 Alternative complement pathway activity

Alternative complement pathway (ACP) activity was measured according to Sunyer and Tort [37] with slight modifications. Several buffers were used: EDTA (ethylene glycol tetraacetic acid)-GVB (Isotonic veronal buffered saline), pH 7.3, containing 0.1% gelatin, 20 mM EDTA, 5 mM Sodium barbiturate, 0.13 mM NaCl; and Mg^+2^-EGTA-GVB, which is GVB with 10 mM Mg^+2^ and 10 mM EGTA instead of EDTA. Rabbit red blood cells (RaRBC; Probiologica Lda, Portugal) were used for natural haemolytic complement determination. RaRBC were washed several times in 0.9 % NaCl and resuspended to a concentration of 2.8×10^8^ cells mL^−1^ in 0.9 % NaCl. 20 µL of RaRBC suspension was then added to 20 µL of serially diluted plasma in Mg-EGTA-GVB buffer. Maximum (100%) and minimum (spontaneous) haemolysis values were obtained by adding 40 µL of distilled water or Mg-EGTA-GVB buffer to 20 µL RaRBC suspension. Samples were incubated at room temperature for 100 min with shaking every 20 min. Subsequently, 150 µL of cold EDTA-GVB buffer was used to stop the reaction. Samples were centrifuged (400 *x g*, 2.5 min, 22 ºC) and the degree of haemolysis was quantified by measuring the OD of the supernatant at 414 nm in a microplate reader (Synergy HT). The degree of haemolysis (Y) was estimated plotting Y (1-Y)^−1^ on a log-log scale graph against the lysis curve for each specimen. The volume of plasma producing 50% hemolysis (ACH_50_) was estimated and the number of ACH_50_ units mL^−1^ was obtained for each sample.

#### 2.6.5 Nitric oxide (NO)

Plasmatic total nitrite plus nitrate content was determined using a Nitrate/Nitrite colorimetric kit (Roche Diagnostics GmbH, Mannheim, Germany, ref: 11,746,081,001) adjusted for 96-well microplates. Briefly, 10 µL of defrosted plasma and 90 µL of distilled water were added to a microplate well. Thereafter, the nitrate reduction into nitrite was accomplished by adding 50 µL of reduced NADPH and 4 μL of the enzyme nitrate reductase and incubating the microplates for 30 min at 25˚C. Subsequently, 50 µL of sulfanilamide and 50 μL of N-(1-naphthyl)-ethylene-diamine dihydrochloride were added and the incubation continued for more 10 min at 25˚C. Then, the OD was measured at 540 nm in a Synergy HT microplate reader. The blank was made using water instead of plasma. Nitrite and nitrate concentrations were determined by comparison with sodium nitrite and potassium nitrate standard curve. As both compounds are oxidative metabolites of endogenously produced NO, they were utilized to quantify NO content in plasma [38].

## 2.7 Bactericidal activity

Two opportunist marine pathogenic bacteria [*Vibrio anguillarum* and *Photobacterium damselae* subsp. *piscicida* strain PP3 (*Phdp*)] were used in the bactericidal activity assay. Bacteria were cultured for 48 h at 25˚C on tryptic soy agar (TSA) in agar plates and then inoculated in tryptic soy broth (TSB, Sigma), both media were supplemented with 1 % NaCl (w/v), and cultured overnight at the same temperature with continuous shaking (100 rpm). Then, bacteria were collected by centrifugation at 3500 *x g* for 30 min and resuspended in sterile HBSS and adjusted to 1×10^6^ colony forming units (cfu) mL^−1^ according to Costas *et al.* [39]. Plasma bactericidal activity was assessed according to Graham *et al.* [40], with slight modifications. Briefly, 20 µL of plasma and 20 µL of both bacteria (1×10^6^ cfu mL^−1^) were added to duplicate wells of U-shaped 96-well plates. Two wells with 20 µL of the bacterial solution and 20 µL of Hank's Balanced Salt Solution (HBSS) served as positive control and two wells with 40 µL of HBSS were used as a negative control. The plates were incubated for 3 h at 25˚C. Then, 25 µL of 2-(4-iodophenyl)-3-(4-nitrophenyl)-5-phenyl-2H-tetrazolium chloride (1 mg mL^−1^; Sigma) was added to each well and incubated for 10 min at 25˚C to enable the formation of formazan. Then, plates were centrifuged (2000 *x g* for 10 min) and the precipitate was dissolved in 200 µL of dimethyl sulfoxide (Sigma). Then, 100 µL of that solution was added to flat-bottomed 96-well plates and the OD measured at 490 nm in a microplate reader (Synergy HT). Bactericidal activity is expressed as the percentage of bacteria that survived relatively to the number of bacteria from the positive controls (100%).

## 2.8 Gene expression

Analyses of mRNA levels were performed in DI samples (6 fish per treatment). Total RNA was extracted using TRIzol reagent (Direct-zolTM RNA Miniprep, Zymo Research) according to manufacturer recommendations, and RNA quality and quantity were assessed by electrophoresis in 1% agarose gel and spectrophotometry (µDrop™ plate, ThermoScientific). The resulting total RNA concentration was adjusted to 0.5 µg/8 µL H_2_0 to complementary DNA synthesis, using the NZY First-Strand cDNA Synthesis Kit (NZYTech, MB12501, Lisbon, Portugal). Gene expression was determined by real-time quantitative PCR (q-PCR) using the Bio-Rad CFX Connect Real-Time System (California, USA). The analysis was carried out using 0.4 µL diluted cDNA (1:2) mixed with 0.2 µL of each primer (10 µΜ), 5 µL of SsoAdvanced™ Universal SYBR® Green Supermix, Bio-Rad Laboratories®, and 4.2 µl DNase/RNase/Protease-free water (Sigma-Aldrich), in a total volume of 10 µL. Primers were obtained from the literature and are shown in Table 2. The efficiency of PCR primers was measured by the slope of a standard curve using serial dilutions of cDNA. Thermal-cycling was initiated with incubation at 95˚C for 30 s for hot-start iTaqΤΜ DNA polymerase activation. A total of forty steps of PCR were then performed, each one consisting of heating at 95˚C for 15 s for denaturing followed by 30 s at 60˚C for annealing and extension. Following the final PCR cycle, melting curves were systematically monitored (65˚C temperature 0.5˚C 10 s^−1^ from 65 to 95). Each PCR run included duplicates of reverse transcription for each sample and negative controls. The PCR run for the reference gene included duplicates for each sample and negative controls. Quantification of the target gene transcripts was performed using elongation factor 1α (*ef1α*) as the reference gene. Relative quantification of the target gene transcript with *ef1α* reference gene transcript was performed using the mathematical model described by Pfaffl [41]. For each mRNA, gene expression was normalized by *ef1α* content in each sample.

**Table 2:** Sequences of the primer pairs used for determination of the transcript level of immune-related genes in the distal intestine in gilthead sea bream.

**Gene**	**Gene abbreviation**	**Primer sequences (5´→3´)**	**Primer efficiency**	**Accession number**
Major histocompatibility complex II	*mhc-IIα*	F:CTGGACCAAGAACGGAAAGA R:CATGGACTCTGAGTAGCGCGA	2.00	DQ019401
Tumor necrosis factor α	*tnfα*	F:TCGTTCAGAGTCTCCTGCAG R:CATGGACTCTGAGTAGCGCGA	2.02	AJ413189
Immunoglobulin M heavy chain	*igm*	F:CAGCCTCGAGAAGTGGAAAC R:GAGGTTGACCAGGTTGGTGT	2.04	AM493677
Cyclooxygenase 2	*cox2*	F:GAGTACTGGAAGCCGAGCAC R:GATATCACTGCCGCCTGAGT	1.92	AM296029
Interleukin 1β	*Il-1β*	F:GGGCTGAACAACAGCACTCTC R:TTAACACTCTCCACCCTCCA	2.05	AJ277166
Proliferating cell nuclear antigen	*pcna*	F:GATGTGGAGCAGCTGGGTAT R:TGTCTACGTTGCTGGTCTGG	1.94	FG263675
Elongation factor 1-alpha	*ef*1*α*	F:CTGTCAAGGAAATCCGTCGT R:TGACCTGAGCGTTGAAGTTG	2.05	AF184170

## 2.9 Statistical analysis

Data are expressed as mean± standard error of the mean (SEM) or standard error (SE). Outliers were removed and normality and homogeneity of data were accessed by the Shapiro-Wilk and Levene tests, respectively, and normalized when appropriate. Statistical evaluation of the data was done by one-way ANOVA. Differences were considered statistically significant at P < 0.05. When p-values were significant, differences between means were evaluated with the Tukey´s test. All statistical analysis was performed using SPSS 24.

## 3 Results

### 3.1 Haematological profile, RBC, WBC, and differential cell counts

After the 56 days of the feeding trial period, dietary treatments did not affect, final weight, weight gain and daily growth index, but diet B lead to the highest feed efficiency and protein efficiency ratio and diet A and D modulated liver lipid content, β-oxidation and lipogenesis (results presented in [29]). Dietary ARA/EPA/DHA ratios did not affect the RBC and WBC numbers, haematocrit (HT), haemoglobin (HB), mean corpuscular volume (MCV), mean corpuscular haemoglobin (MCH), and mean corpuscular haemoglobin concentration (MCHC) (Table 3). In the case of differential cell counts, no variations in the differential WBC numbers were observed, except for monocytes numbers which were higher in fish fed diet B than fish fed diet C whilst no variations were found respect to values found in the fish fed other experimental diets (A and D) (Table 3).

**Table 3:** Haematological profile in gilthead sea bream fed the experimental diets (A, B, C, D) after 56 days.

Parameters	Dietary treatments (ARA/EPA/DHA ratio)				SEM
	A	B	C	D	
	2.0/0.2/0.1	1.0/0.4/0.4	0/0.6/0.6	0/0.3/1.5	
Haemoglobin (g dL)	3.7	4.1	3.6	3.4	0.1
Haematocrit (%)	29.1	30.9	28.4	29.8	0.5
MCV (µm^3^)^1^	200.6	220.6	198.9	209.5	6.2
MCH (pg cell^−1^)^2^	25.9	29.2	24.5	23.8	1.2
MCHC (g 100 mL^−1^)^3^	12.9	13.4	13.1	11.0	0.5
RBC (x 10^6^ µL^−1^)	1.5	1.4	1.4	1.5	0.0
WBC (x 10^4^ µL^−1^)	6.9	6.7	7.1	7.5	0.2
Neutrophils (10^4^ µL^−1^)	0.38	0.50	0.36	0.48	0.0
Monocytes (10^4^ µL^−1^)	0.25^ab^	0.29^b^	0.14^a^	0.15^ab^	0.0
Lymphocytes (10^4^ µL^−1^)	0.60	0.69	0.84	0.92	0.1
Thrombocytes (10^4^ µL^−1^)	5.4	5.2	5.7	6.0	0.2

Values presented as means (n=9) and standard error of the mean (SEM). Superscript letters indicate significant differences between dietary treatments (P < 0.05). MCH: mean corpuscular haemoglobin; MCHC: mean corpuscular haemoglobin concentration; MCV: mean corpuscular volume; RBC: red blood cells; WBC: white blood cells.

### 3.2 Humoral immune parameters

Innate immune parameters evaluated, namely peroxidase, antiprotease, protease, and nitric oxide, were not affected by any dietary treatment as is summarized in Table 4. Contrarily, the haemolytic complement activity increased in the plasma of fish fed diet B compared to values found in fish fed diet D, whereas no significant differences were observed compared to values registered in the other experimental groups (Table 4).

**Table 4:** Immune humoral parameters measured in gilthead sea bream juveniles plasma fed the experimental diets (A, B, C, D) after 56 days.

Parameters	Dietary treatments (ARA/EPA/DHA ratio)				SEM
	A	B	C	D	
	2.0/0.2/0.1	1.0/0.4/0.4	0/0.6/0.6	0/0.3/1.5	
Peroxidase activity (U mL^−1^)	33.2	57.8	58.6	81.7	8.4
Nitric oxide (µM)	986.6	1000.4	1107.3	1069.8	24.2
Antiprotease activity (%)	86.3	86.8	86.7	86.3	0.1
Protease activity (%)	7.3	7.3	7.2	7.0	0.1
Alternative complement pathway activity (ACH_50_ U mL^−1^)	12.6^ab^	15.6^b^	13.4^ab^	9.9^a^	0.6

Values are presented as means (n=9) and standard error of the mean (SEM). Superscript letters indicate significant differences between dietary treatments (P < 0.05).

### 3.3 Bactericidal activity

**Fig. 1:**
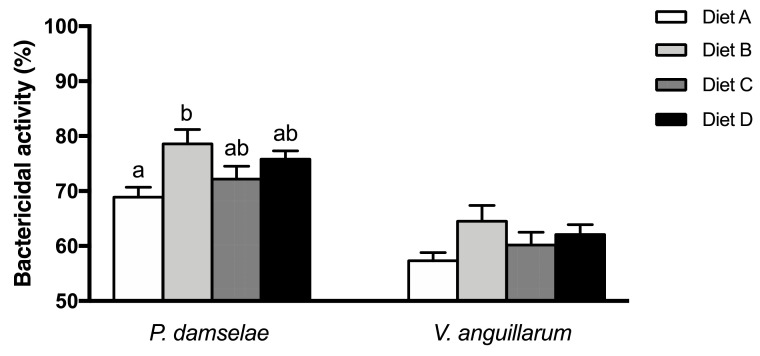
Bactericidal activity (%) against *Photobacterium damselae* subsp. *piscicida* and *Vibrio anguillarum* in the plasma of gilthead sea bream juveniles fed the experimental diets (A, B, C, D) after 56 days. Data represent the mean ± standard error (SE) (n=9). Different letters denote significant differences between experimental groups (P < 0.05).

Bactericidal activity against two pathogenic bacteria was measured in the plasma of gilthead sea bream fed experimental diets (Fig. 1). In the case of plasma bactericidal activity against *Phdp*, the values increased in fish fed diet B compared to fish from group A, whilst the activity not varied in fish fed diets C and D (Fig. 1). Contrarily, the bactericidal activity against *V. anguillarum* was not affected in the plasma of fish from any dietary treatment (Fig. 1).

### 3.4 Immune-related gene expression in the intestine

**Fig. 2:**
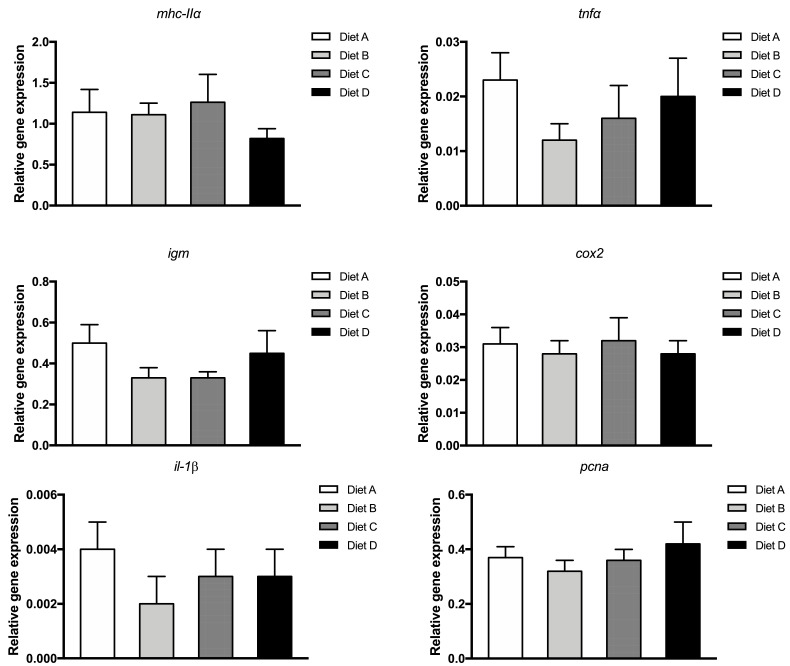
Gene expression in the distal intestine of gilthead sea bream fed the experimental diets (A, B, C, D) after 56 days. Immune-related gene expression detected by rt-PCR and normalized to housekeeping gene elongation factor 1-alpha (*ef1α*). Data represent the mean ± standard error (SE) (n=6).

At the end of the feeding trial, no significant differences were observed in the gene expression of major histocompatibility complex II (*mhc-IIα*), tumor necrosis factor α (*tnfα*), immunoglobulin M heavy chain (*igm*) and proliferating cell nuclear antigen (*pcna*) in the intestine of fish fed experimental diets (Fig. 2). Similarly, in the case of expression of cyclooxygenase 2 (*cox2*) and interleukin 1β (*il-1β*) genes, both related to inflammatory processes, no significant variations were observed in the intestine of any experimental group (Fig. 2).

## 4.Discussion

The n-3 LC-PUFA requirements for juvenile gilthead sea bream (average body weight of 42 g) were estimated to be 0.9 % DM with an optimum DHA/EPA ratio of 1 [9]. The ARA requirements for gilthead sea bream have not yet been determined. Accordingly, Diet B was designed to match the minimum requirements of ARA, EPA, and DHA for gilthead sea bream juveniles and represents a balance diet of the study with similar ARA and EPA+DHA levels dietary content (ARA, 1%DM= EPA, 0.4%DM + DHA, 0.4%DM; DHA/EPA=1). Diet A was formulated with an extreme ARA level (2% DM) and almost devoid of EPA (0.2%DM) and DHA (0.1%DM). Diet C was formulated to be devoid of ARA and rich in EPA +DHA (0.6+0.6 % DM; 1:1 ratio) and diet D was formulated with high levels of n-3 LC-PUFA (1.8%DM) but with an unbalance DHA:EPA ratio of 5:1 (DHA, 1.5% DM; EPA, 0.3% DM) and without ARA.

The dietary EFA composition also affects the lipid composition of RBC and WBC and cell function by impacting fluidity [42] and fragility [17]. In the present study, after 56 days of feeding the gilthead seabream juveniles with the experimental diets, no differences were detected in the numbers of RBC and WBC in the blood of fish. These results are in agreement with previous data from gilthead sea bream where low dietary n-3 LC-PUFA (0.42 %) level did not affect the values of RBC numbers and HT at low stocking densities, while at high stock densities, the number of RBC was reduced compared to fish fed high n-3 LC-PUFA (1.5 %) diet [21]. Results are however inconsistent and in a more recent study with gilthead sea bream, the RBC numbers were increased in fish fed a diet low in n-3 LC-PUFA (0.5 %) compared to groups fed the control diet with 2% n-3 LC-PUFA [17]. Similarly, n-3 LC-PUFA deficient diets also did not affect the RBC numbers, HB, and HT in rockfish (*Sebastes schlegeli*) [43] and Japanese eel (*Anguilla japonica*) fed increasing dietary ARA levels from 0 to 1.65% [44]. Contrarily, for other species, reduction of dietary n-3 LC-PUFA levels through the replacement of FO by a VO reduced the values of HT and HB in adult cod (*Gadus morhua*) [45] and RBC numbers in turbot (*Scophthalmus maximus*) [46]. Moreover, contrary to present results, reduction of dietary DHA/EPA ratio (0.9) decreased the RBC numbers and HT values and consequently increased MCV and MCH values in silvery-black porgy (*Sparidentex hasta*) [47].

In the case of leucocytes, the WBC numbers were also not affected by dietary ARA or n-3 LC-PUFA levels in Japanese eel [44], silvery-black porgy [48], channel catfish, (*Ictalurus punctatus*) [49], Atlantic salmon (*Salmo salar*) [50], and black seabream (*Acanthopagrus schlegelii*) [51], while in Indian major carp (*Catla catla*), the increase of dietary n-3 LC-PUFA decreased the WBC numbers [52]. Concerning differential counts of WBC, the number of monocytes decreased in fish fed diets C and D. Interestingly, these diets, had not been supplemented with ARA. This could suggest that adequate dietary ARA content is necessary for proper monocytes recruitment. The lack of effect of diet A which only had 0.3% n-3 LC-PUFA contrasts that of Montero *et al.* [17] who found reduced lymphocytes numbers in gilthead sea bream when fed 0.5 % n-3 LC-PUFA content. This could indicate that elevated ARA levels may compensate for some of the n-3 LC-PUFA deficiency. Further, in Atlantic salmon and channel catfish, diets with high n-3 LC-PUFA levels increased the thrombocytes numbers [49, 50]. This was not seen in the present experiment, but there was a small but notable increase in thrombocytes in fish fed high n-3 LC-PUFA (diets C and D).

Previously, it was been shown that deficiency of n-3 LC-PUFA leads to depletion of several innate immune responses including haemolytic complement activity in gilthead sea bream [17, 21]. On the other hand, also in gilthead sea bream, elevated levels of n-6 FA along with reduced levels of EPA and DHA have also been shown to decrease serum bactericidal activity [53]. In the present study, the results clearly suggest that balanced ARA, EPA, and DHA levels are required for optimum immune capacity in gilthead sea bream. Compared to the other diets, diet B (1.0/0.4/0.4, ARA/EPA/DHA, respectively) had elevated haemolytic complement activity and bactericidal response against *Phdp* compared to the values found in fish fed other experimental diets [54]. As is known, bactericidal activity measures the capability of plasma to kill a bacteria and is considered one of the most practical immune parameters to assess the general health status of the fish as it includes the activity of several plasmatic compounds [55]. However, in the present study, deficiency in the n-3 LC-PUFA (Diet A) clearly impaired the bactericidal activity against *Phdp* but no diet influenced the response against *V. anguillarum*. This fact could be related to the different virulence mechanisms of each pathogenic bacterium. The virulence of *Phdp* is associated with a polysaccharide capsular layer [56] while the virulence of *V. anguillarum* is linked with exopolysaccharides secreted into the surrounding environment that increase resistance to host lysozyme and antimicrobial peptide activities [57] and that may protect more effectively the bacterial cell wall.

Despite numerous studies on the potential influence of dietary EFA on fish immunity the underlying mechanisms on how ARA, EPA, or DHA affects these responses remain unclear [16, 44, [58], [59], [60], [61], [62], [63], [64], [65]]. An imbalance of eicosanoid production is probably central in innate immune responses [16]. Indeed, the balance of eicosanoids production with anti- or pro-inflammatory properties is largely determined by the ARA and EPA ratio in cellular membranes [66]. ARA is the precursor of pro-inflammatory and EPA is a precursor of anti or less-inflammatory prostaglandins and leukotrienes [25]. In recent years, several studies have attributed the immunostimulant effect of dietary n-3 LC-PUFA to DHA, and high DHA/EPA ratios have been shown to promote lysozyme and haemolytic complement activity [61], phagocytic and respiratory burst activities in head-kidney leucocyte (HKLs) [65], serum IgM levels and lysozyme activity [58, 64], that was not observed in the present study probably due to the extremely high DHA/EPA ratio (5:1) compared to the ratio used in the mentioned studies or absence of inflammatory response.

While it is generally given more relevance to n-3 LC-PUFA as EFA in marine fish, as growth performance is mainly depressed when they are included at low levels in the diets, an adequate dietary ARA level is also relevant when the immune status is considered, as shown in the present study. The importance of dietary ARA was also shown in European seabass, as increasing dietary levels from 0.5 to 1% improved HKLs phagocytic capacity [16]. Furthermore, in Japanese eel, giving 1.06 % ARA increased the serum lysozyme activity compared to those fed 0 or 0.33 % ARA diets [44]. Also, in juvenile Japanese seabass (*Lateolabrax japonicas*)*,* modest dietary ARA levels of 0.36 and 0.56 % enhanced serum lysozyme and haemolytic complement activity compared with fish fed 0.08 % diet [60]. Furthermore, though not statistically different, increasing dietary ARA (0.05–0.65%) content improved immune parameters, HKLs respiratory burst, and serum lysozyme activity in Malabar red snapper (*Lutjanus malabaricus*) [67].

The intestine is one of the main entrances for pathogens if its integrity is compromised [27], and this may occur by using alternative-feedstuffs based diets. However, in the present trial, distal intestine histomorphology was not compromised by the dietary treatments (results presented in [68]). Also, the DI cellular content of ARA, EPA, and DHA determined the pattern of eicosanoids produced by cyclooxygenases and lipoxygenases [69]. ARA derived eicosanoids are, generally, associated with a potent inflammatory effect via PGE_2_ due to the production and release of proinflammatory cytokines such as TNFα and IL-1β [70]. Contrary, eicosanoids produced from EPA such as leukotriene 5 (LTB_5_), and PGE_3_ are less potent and frequently associated with anti-inflammatory properties by decreasing the expression of inflammatory cytokines [24]. However, the different dietary EFA ratios of the present study did not affect cytokines gene expression, *tnfα,* and *il-1β,* which are proteins produced by activated immune-related cells such as monocytes and lymphocytes, and modulate the immune response [71]. The lack of effects in immune-related genes expression in the DI reveal significant robustness of the intestine of gilthead sea bream and a possible lack of inflammatory response. Previously in gilthead sea bream, it was also observed that replacement of FO by soybean or linseed oils did not affect the intestinal expression of *tnfα* and *il-1β* genes whilst in the HK was observed an up-regulation of *tnfα* gene expression [28], suggesting different responses according to the tissues.

Contrary to the present results, 0.5 and 1 % dietary ARA was associated with changes in the expression of GALT-related genes in European seabass, increasing the gene expression of *il-1β* and the anti-inflammatory cytokine *il-10* [72]. However, 2 days post-infection with *V. anguillarum*, 2 % of ARA increased the expression of *il-1β* and *cox2* genes and 4 % increased *il-10* expression [72]. Furthermore, in the anterior intestine of gilthead sea bream, dietary inclusion of modified *Camelina sativa* oil with 2.4 % of EPA and DHA down-regulated the expression of the *il-8* gene compared to the control diet [73]. In the current work, different dietary n6/n3 ratios did not affect the expression of the *cox2* gene in the DI implying no effect on PGE_2_ production, as described recently in European seabass by Rivero-Ramírez *et al.* [72]. However, a preference for released ARA instead of EPA by the COX2 enzyme as substrate was already described in brook trout (*Salvelinus fontinalis*) [74] and Atlantic cod (*Gadus morhua*) [75]. The authors suggested an evolutionary adaptation of the enzyme to the scarce amounts of ARA in the marine system leading to a strong preference for this EFA compared to EPA or other n-3 fatty acids available [74]. The lack of dietary effect on the expression of the *cox2* gene of DI may, at least in part, be attributed to relatively low ARA levels of intestine tissue, irrespective of the dietary ARA levels. Indeed, in the present study, the muscle ARA levels of gilthead sea bream fed the experimental diets, were maintained relatively constant despite the increase of dietary ARA level [29], and a similar intestinal FA profile is expected.

For Senegalese sole (*Solea senegalensis*), Montero *et al.* [76] also showed that FO substitution by linseed oil did not affect the intestinal *cox2* gene expression levels whereas with soybean oil the expression levels increased. The result can not be explained by the ARA/EPA ratio of intestine FA composition because it was similar [76]. Instead, the explanation of the results may be associated with the n-3/n-6 ratio that was lower in fish fed soybean oil diet compared to the control diet mainly due to the higher linoleic acid (18:2n-6) content in linseed oil [76]. It has also been suggested, for gilthead sea bream, that ARA mediated effects could not always be attributed to an increase in prostaglandin synthesis by cyclooxygenase. Indeed, in gilthead sea bream juveniles submitted to stress, it was observed higher plasma cortisol levels in fish fed high ARA dietary content and acetylsalicylic acid (ASA), an irreversible blocker of cyclooxygenase, than fish fed high ARA diets without ASA [77]. Nonetheless, in Atlantic cod HKLs, the *cox2* mRNA expression seems to be correlated with PGE_2_ production but not with PGE_3_ [75]. Furthermore, increasing concentrations of EPA and ARA in equal amounts showing the preference of *cox2* by ARA as substrate but similar to the presented results, the addition of ARA alone increased PGE_2_ secretion but did not induce *cox2* transcription [75].

The dietary EFA ratio also did not modulate the gene expression in the distal intestine of major histocompatibility complex (*mhc-IIα*), which has a pivotal role in immune response activation and cellular markers produced by B-cells (*igm*). Furthermore, some shreds of evidence suggested that EPA and DHA content of fish oil might decrease *mhc-IIα* expression consequently decreasing the acquired immunity to a pathogen [78]. The presented results suggested a lack of acquired immunity response. The *mhc-IIα* is a key molecule to initiate the T cell-mediated adaptive response through the action of antigen-presenting cells like macrophages or B-cells [79, 80]. Similarly, also in gilthead sea bream, intestinal *igm* expression was not affected by the dietary substitution of 66 % FO by a blend of VOs [81]. Also with dietary inclusion of microalgae, which are rich in EFA, namely EPA, minor dietary influence on the expression of *mhc-IIα* and *igm* genes was reported in HK and intestine of gilthead sea bream [82, 83].

In conclusion, results of the present study indicate that diets with balanced dietary ARA, EPA, and DHA (1/04/0.4% DM) level increased circulating monocytes numbers, haemolytic complement and bactericidal activity against *Phdp*, thus improving the immunological status of gilthead sea bream juveniles under unchallenged conditions. Furthermore, no differences in the immune-related genes evaluated in the distal intestine were found between different ARA/EPA/DHA levels. However, further studies will be necessary to further evaluate the effects of the dietary EFA ratios under challenging conditions.

## Data availability

The data generated during and/or analysed during the current study are available from the corresponding author on reasonable request.

## Declaration of Competing Interests

The authors declare that they have no known competing financial interests or personal relationships that could have appeared to influence the work reported in this paper.
